# Evaluating global and local sequence alignment methods for comparing patient medical records

**DOI:** 10.1186/s12911-019-0965-y

**Published:** 2019-12-19

**Authors:** Ming Huang, Nilay D. Shah, Lixia Yao

**Affiliations:** Department of Health Sciences Research, Mayo Clinic, Rochester, MN USA

**Keywords:** Patient similarity, Electronic health record, Sequence alignment, Temporal sequence, Dynamic time warping, Needleman-Wunsch algorithm, Smith-Waterman algorithm

## Abstract

**Background:**

Sequence alignment is a way of arranging sequences (e.g., DNA, RNA, protein, natural language, financial data, or medical events) to identify the relatedness between two or more sequences and regions of similarity. For Electronic Health Records (EHR) data, sequence alignment helps to identify patients of similar disease trajectory for more relevant and precise prognosis, diagnosis and treatment of patients.

**Methods:**

We tested two cutting-edge global sequence alignment methods, namely dynamic time warping (DTW) and Needleman-Wunsch algorithm (NWA), together with their local modifications, DTW for Local alignment (DTWL) and Smith-Waterman algorithm (SWA), for aligning patient medical records. We also used 4 sets of synthetic patient medical records generated from a large real-world EHR database as gold standard data, to objectively evaluate these sequence alignment algorithms.

**Results:**

For global sequence alignments, 47 out of 80 DTW alignments and 11 out of 80 NWA alignments had superior similarity scores than reference alignments while the rest 33 DTW alignments and 69 NWA alignments had the same similarity scores as reference alignments. Forty-six out of 80 DTW alignments had better similarity scores than NWA alignments with the rest 34 cases having the equal similarity scores from both algorithms. For local sequence alignments, 70 out of 80 DTWL alignments and 68 out of 80 SWA alignments had larger coverage and higher similarity scores than reference alignments while the rest DTWL alignments and SWA alignments received the same coverage and similarity scores as reference alignments. Six out of 80 DTWL alignments showed larger coverage and higher similarity scores than SWA alignments. Thirty DTWL alignments had the equal coverage but better similarity scores than SWA. DTWL and SWA received the equal coverage and similarity scores for the rest 44 cases.

**Conclusions:**

DTW, NWA, DTWL and SWA outperformed the reference alignments. DTW (or DTWL) seems to align better than NWA (or SWA) by inserting new daily events and identifying more similarities between patient medical records. The evaluation results could provide valuable information on the strengths and weakness of these sequence alignment methods for future development of sequence alignment methods and patient similarity-based studies.

## Background

Patient similarity calculation has become an emerging research topic. It identifies similar patients in a large pool for healthcare insights on prognosis, diagnosis, and treatment [1, 2]. For example, in case of a patient with a rare or hard-to-diagnosed disease, identifying patients with similar disease trajectory might expedite the diagnosis and treatment and reduce patient suffering. In addition, patient similarity calculation is critical to machine learning based prediction tasks such as disease prognosis, medication outcomes and mortality [3, 4]. We refer the readers to a few review papers for patient similarity calculation and its implications for precise medication [4–6].

**Fig. 1:**
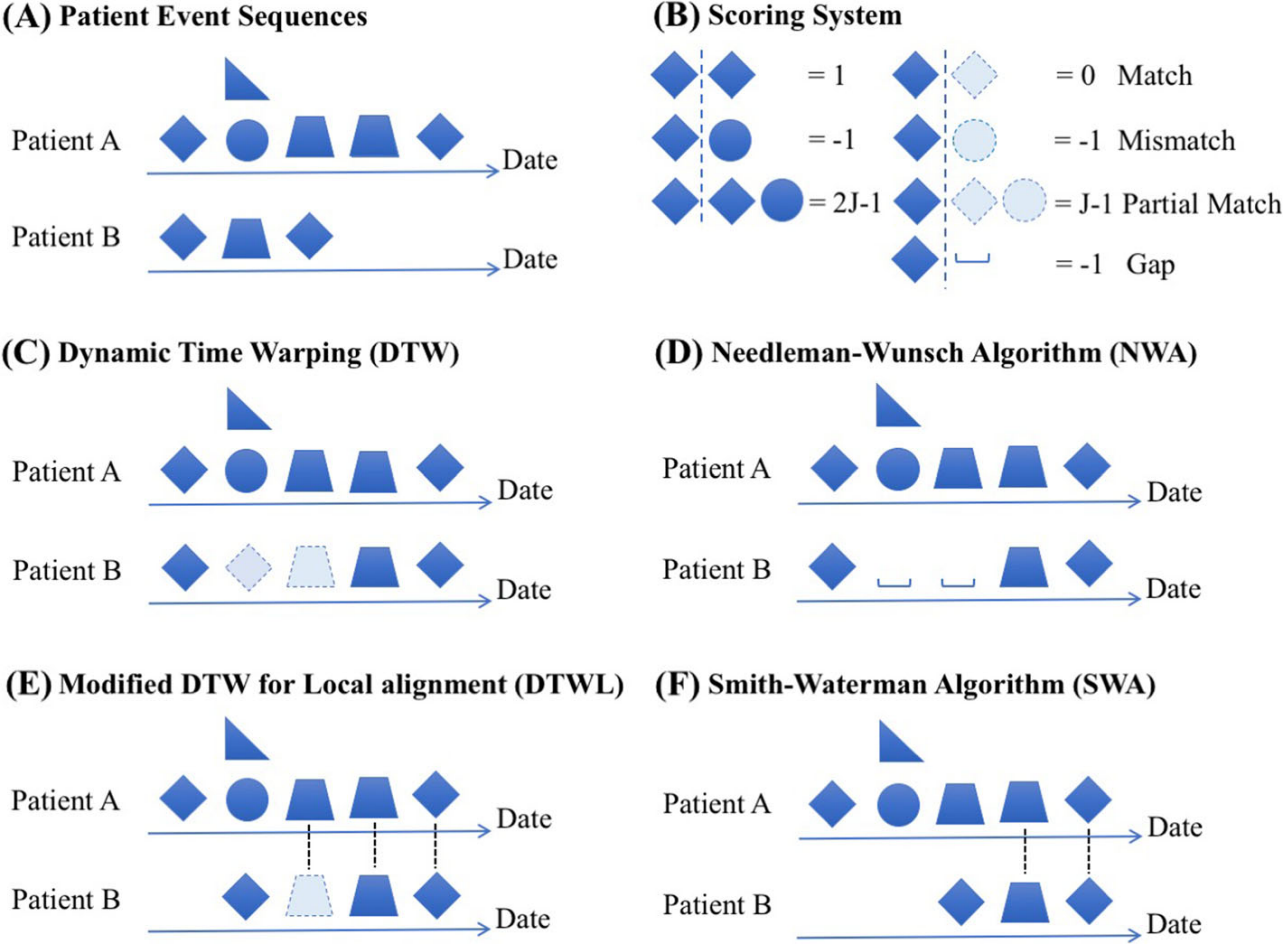
An illustration demonstrating the significance of sequence alignment. (**A**) Two simplified temporal event sequences; (**B**) the scoring function to calculate the pairwise patient similarity; global sequence alignment algorithms, DTW (**C**) and NWA (**D**); local sequence alignment algorithms, DTWL (**E**) and SWA (**F**). The shapes with light blue and dash border are extra medical events inserted by DTW or DTWL during sequence alignment. “_” is a gap spot inserted by NWA or SWA during sequence alignment. The different shapes (e.g., diamond, triangle and circle) represent different medical events. J denotes Jaccard index

When calculating and comparing patient similarity from electronic health records (EHRs) data, we could not bypass the issue of aligning the temporal event sequences [7]. Mathematically and computationally, EHR of a patient can be viewed as a temporal sequence of medical events. As illustrated in Fig. 1(A), patient A and patient B do not look similar without properly alignment first. Figure 1 (C), (D), (E) and (F) demonstrate different strategies to align the temporal event sequences of two patients. Patient similarity calculation with proper sequence alignment suggests a novel solution to reserve temporal information in EHRs [8, 9]. Che et al. for first time deployed dynamic time warping (DTW) to align temporal sequence when calculating patient similarity. They adopted a linear regression model with a subset of patients that are most similar to a target patient and achieved a better F1 score (77%) at predicting the target patient’s Parkinson subtype, compared to the same model using all patients (75%) [8].

DTW is a global sequence alignment method based on dynamic programming. It finds an optimal match between two sequences of feature vectors by stretching and/or compressing one or more sections of one sequence and is considered as the best alignment method for various applications including speech recognition and video streaming [8]. Sequence alignment is also extensively used in bioinformatics, in particularly at comparing protein, DNA or RNA sequences to identify regions of similarity that may be a consequence of functional, structural or evolutionary relationships between the sequences. Needleman-Wunsch Algorithm (NWA) is a widely used global alignment algorithm for aligning protein, DNA or RNA sequences [10, 11]. In contrast to DTW which stretches a section of a sequence and fills in missing events with the adjacent event, NWA pads the missing events with gap as direct and deterministic penalization. Besides global sequence alignments, local sequence alignments are more useful to identify the similar sequence motifs among not so similar sequences. Smith-Waterman Algorithm (SWA) is a variation of NWA for local sequence alignments [12]. SWA is broadly used for determining similar regions between two nucleic acid sequences or protein sequences [13, 14].

Considering the significance of temporal information in medicine, we are curious to ask the question – which type of sequence alignment method works best for EHR data? Unfortunately, no objective and comprehensive evaluation and comparison between state-of-art sequence alignment methods is available. Therefore, we plan to compare the strengths and limitations of both global and local sequence alignment methods and evaluate their impact on patient similarity calculation. This is a challenging task for several reasons:

Firstly, patient medical records are complex [15–17]. We use the most structured and standardized EHR data type – diagnosis to illustrate. There are thousands of diagnosis codes, whereas DNA sequences have only four types of nucleic acids and protein sequences contain 20 types of amino acids. All the diagnosis codes are documented in EHR in the same way, but their semantic meaning can be very different. For instance, a diagnosis code of diabetes on a certain date does not mean diabetes only occurs at that specific time point. Medically speaking, diabetes is not curable. Once a person is diagnosed with diabetes, he or she will carry diabetes for the rest of the life. Only under successful management, diabetes can go into “remission” state. However, influenza is more of an acute condition that patient can recover from in a short period of time. The data quality also varies. Some patients have a few lines on their medical records, whilst others have thousands of lines attributed to many clinical encounters. A long period of gap in patient medical records can mean either “healthy state” or missing. Such ambiguity is hard to resolve without further information. Other EHR data types, such as medications, procedures, lab tests, and clinical notes are no less complicated.

Secondly, no gold standard data is available for evaluating sequence alignment algorithms. One solution is to ask experts, such as physicians to evaluate and rank the results from different sequence alignment methods, which can be very subjective and expensive. In this work, we propose to synthesize simulated patient medical records using seed patients carefully chosen from a large real-world EHR database. We will be able to design and control the differences between sequences of medical records for objective and comprehensive evaluation of sequence alignment algorithms.

The rest of the paper is organized as the following 5 sections. In the Related Work section, we will describe three global and local sequence alignment algorithms, namely DTW, NWA and SWA. In the Methods section, we will introduce the methods for selecting seed patients from a large real-world EHR database and for synthesizing more patient medical records; the implementation of DTW, NWA, SWA and DTWL, a modified DTW for local alignment; and the metrics for evaluating sequence alignment results. In the Results section, we will share and analyze briefly the alignment results. In the Discussion section, we will evaluate these sequence alignment methods in details and illustrated various scenarios of sequence alignments using simplified cases. We also discuss the limitations of our work. In the end we will conclude our work.

## Related work

***Dynamic Time Warping (DTW)*** is one of the leading matching algorithms for globally aligning two temporal sequences of different speeds and measuring similarity [8, 18]. Specifically DTW determines the optimal alignment between two given temporal sequences based on the following restrictions and rules:

- Every index in one sequence must match one or more indices in the other sequence. The 1-to-n or n-to-1 index matching denotes the warping in the time dimension.
- The first indices in the two sequences must match.
- The last indices in the two sequences must match.
- The mapping of the indices in the two sequences must be monotonically increasing.

Given two temporal event sequences of two patients ***X*** ([X_1_, X_2_, …, X_i_, …, X_n_]) and ***Y*** ([Y_1_, Y_2_, …, Y_j_, …, Y_m_]), DTW calculates an accumulated score matrix ***A***_*(n + 1) x (m + 1)*_ by updating the matrix element A_i, j_ according to the following equation,

1 $$ {A}_{i,j}=\Big\{{\displaystyle \begin{array}{cc}0& i=0,j=0\\ {}-\infty & i=0,j>0\\ {}-\infty & i>0,j=0\\ {}\max \left(s\left({X}_i,{Y}_j\right)+{A}_{i-1,j-1},s\left({X}_i,{Y}_j\right)+{A}_{i-1,j},s\left({X}_i,{Y}_j\right)+{A}_{i,j-1}\right)& i>0,j>0\end{array}}\operatorname{}\kern0.5em $$

where s (X_i_, Y_j_) denotes the distance between two elements X_i_ and Y_j_ in the sequence of ***X*** and ***Y***. In our experiment, we define s (X_i_, Y_j_) according to the scoring system shown in Fig. 1(B).

DTW then tracks back from the matrix element A_(n + 1), (m + 1)_ to find the optimal alignment path by maximizing the accumulated score in the accumulated score matrix.

***Needleman-Wunsch Algorithm (NWA)*** was firstly developed by Saul B. Needleman and Christian D. Wunsch in 1970 [10]. It was one of the first application of dynamic programming to align and compare protein and nucleotide sequences. As a global alignment method, NWA introduces a gap rather than warping and filling in an adjacent element when aligning sequences. Therefore, every index in one sequence matches another index or a gap in the other sequence, and the monotonic increase of the mapping indices is maintained.

Mathematically, given two temporal sequences of medical events ***X*** ([X_1_, X_2_, …, X_i_, …, X_n_]) and ***Y*** ([Y_1_, Y_2_, …, Y_j_, …, Y_m_]), NWA calculates an accumulated score matrix ***A***_*(n + 1) x (m + 1)*_ by updating the matrix element A_i, j_ according to the following equation,

2 $$ {A}_{i,j}=\Big\{{\displaystyle \begin{array}{cc}0& i=0,j=0\\ {}j\ast gp& i=0,j>0\\ {}i\ast gp& i>0,j=0\\ {}\max \left({A}_{i-1,j-1}+s\left({X}_i,{Y}_j\right),{A}_{i-1,j}+\mathrm{gp},{A}_{i,j-1}+\mathrm{gp}\right)& i>0,j>0\end{array}}\operatorname{} $$

Where gp stands for a gap penalty; s (X_i_, Y_j_) denotes the simialrity between two elements X_i_ and Y_j_ in the sequence of ***X*** and ***Y***, and is calculated using a scoring system shown in Fig. 1(B).

NWA also identifies an optimal alignment path relative to a given scoring system including gap penalty by tracking back from the matrix element A_(n + 1), (m + 1)_ and maximizing the accumulated scores along the path.

***Smith-Waterman Algorithm (SWA)*** is a local sequence alignment algorithm developed by Temple F. Smith and Michael S. Waterman in 1981 [12], which is a variation of NWA for local sequence alignment. SWA has been commonly used for aligning biological sequence, such as DNA, RNA or protein sequences [13, 14].

Given two temporal sequences of medical events ***X*** ([X_1_, X_2_, …, X_i_, …, X_n_]) and ***Y*** ([Y_1_, Y_2_, …, Y_j_, …, Y_m_]), SWA calculates an accumulated score matrix ***A***_*(n + 1) x (m + 1)*_ by updating the matrix element A_i, j_ according to the following equation,

3 $$ {A}_{i,j}=\Big\{{\displaystyle \begin{array}{cc}0& i=0\mathrm{or}j=0\\ {}\max \left({A}_{i-1,j-1}+s\left({X}_i,{Y}_j\right),{A}_{i-1,j}+\mathrm{gp},{A}_{i,j-1}+\mathrm{gp},0\right)& i>0,j>0\end{array}}\operatorname{} $$

Where gp stands for a gap penalty; s (X_i_, Y_j_) denotes the similarity between two elements X_i_ and Y_j_ in the sequence of ***X*** and ***Y***, and is calculated using a scoring system shown in Fig. 1(B).

The main difference from NWA is that the matrix element with negative accumulated score is set to zero, which is used to mask certain mismatched alignments and render locally matched alignments visible. Sequentially, by starting at the element with the highest accumulated score, the algorithm identifies the local alignment path with the highest similarity by tracking back and choosing the path affiliated with maximal accumulated score until the matrix element with an accumulated score of zero is encountered. The algorithm is also guaranteed to find the optimal local alignment with respect to the predefined scoring system.

## Methods

### Real-world EHR database

The Rochester Epidemiology Project (REP) was established in the mid-1960s by Dr. Leonard T. Kurland [19–21]. In 2016, the REP contained approximately 2 million patient records from 54 different health care providers that matched to more than 577,000 individuals who had been residents of Olmsted County at some point between 1966 and 2016. The REP includes demographic data and comprehensive coded information about medical diagnoses, hospital admissions, surgical procedures, prescriptions, laboratory test results, and smoking and body mass index information. Thus it contains complete patient medical records from their outpatient (office visit, urgent care, emergency room) to hospitalization contacts across all local medical facilities, regardless of where the care was delivered or of insurance status. Investigators are able to conduct long-term, population-based studies of disease incidence, prevalence, risk and protective factors, outcomes, health services utilization, and cost-effectiveness. The version of REP database we used in this project is a cut from original database with all patient medical records for the period of 1995–2015.

Without loss of generality, we only considered diagnosis information in this project. This is because all other information in EHRs, such as medications, procedures, lab tests, and clinical notes have dependency on diagnoses. No medications, procedures, lab tests and clinical notes can be easily synthesized to meaningfully simulate real world situations, without considering their dependency on diagnoses and the underlying medical rational.

REP database uses the International Classification of Diseases, Ninth Revision, Clinical Modification (ICD-9-CM) [22] to code diagnosis. ICD-9-CM has refined coding granularity to classify and group diseases and medical conditions and has been primarily used for billing purpose in the United States. As our purpose in this project is to evaluate various sequence alignment approaches for patient similarity calculation and predictive modeling, we first aggregated the ICD-9-CM codes to the PheCode [23]. PheCode represents a granularity of disease concepts that is closer to clinical practice and has proven to have better performance in various data mining tasks [24–26]. We further grouped the diseases defined by PheCode using the digits before the period (.) of PheCode to capture broader disease categories. For example, the code “195.1” was chunked to “195” and consolidated into the “195” category. In total, 14,335 diseases and medical conditions defined by ICD-9-CM in the REP database were grouped into 582 diseases and medical conditions.

### Synthesis of patient medical records

#### Selection criteria of patient medical records

**Fig. 2:**
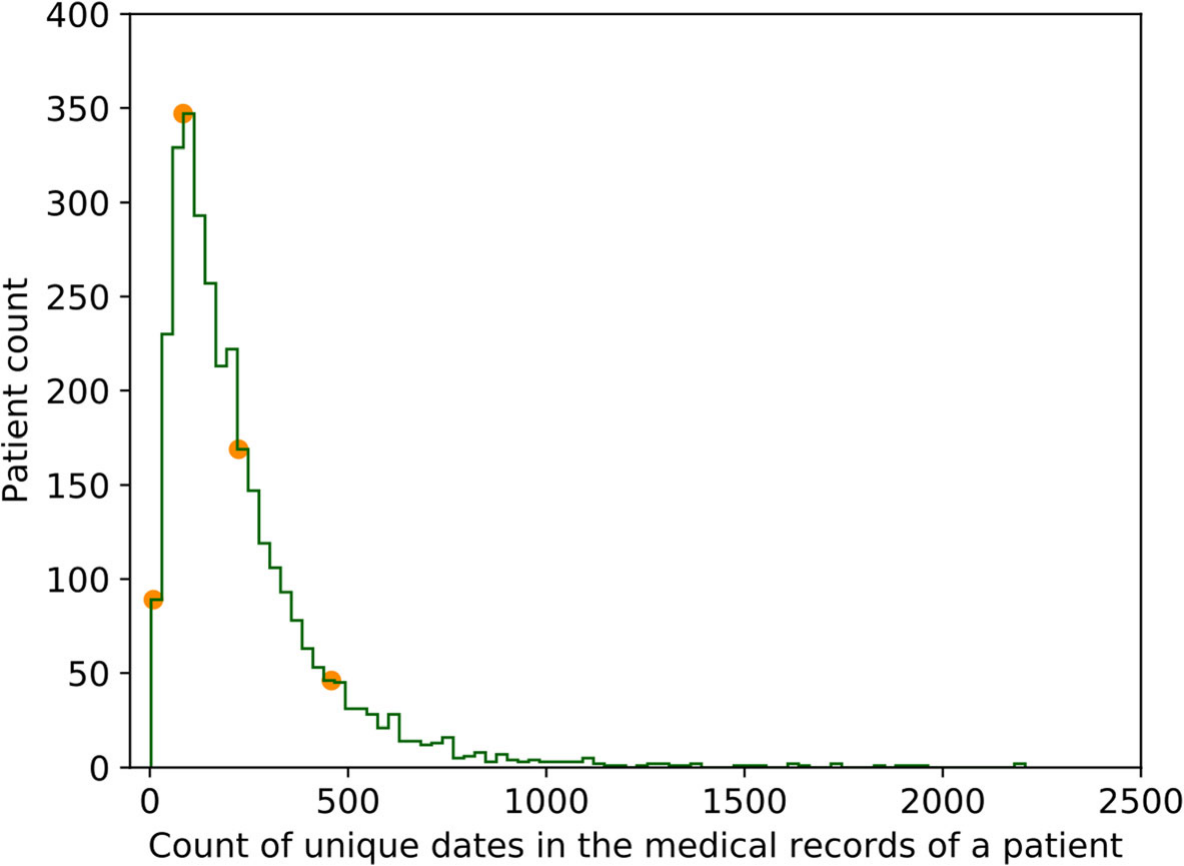
The distribution of medical record length in terms of count of unique dates for patients with influenza (acute disease) and type II diabetes (chronic disease), and with three or more types of clinical encounters on a single day (specified in Table 1) in the REP database

Medical care is highly specialized, complicated and heterogenous. As shown in Table 1, on a single day, a patient may have one or more visits to a clinic, a hospital or other types of healthcare facilities. The diagnosis information observed in most EHR databases would be a list of diagnosis codes for a given date without specifying the order of the events, which we call a daily event. In order to sample representative patients from the REP database and synthesize patient medical records that simulate real world situations, we consider the following characteristics of patient medical records:

1. Multiple scenarios of patient clinical encounters on a single day, including the number of visits per day (single vs. multiple) and the number of diagnosis in each encounter (single vs. multiple);
2. The nature of diseases. Acute diseases on patient medical records can be considered as an event on a specific time point, whereas chronic diseases cover a longer time span. It is hard to infer or reconstruct the time span for each disease in patient medical records without medical knowledge;
3. The lengths of medical records of different patients vary significantly (See Fig. 2). Relocation, job and medical insurance plan changes all impact the lengths of patient medical records.

**Table 1:** Different scenarios of patient clinical encounters on a single day

Daily event		Medical scenario	Diagnosis record^a^
Single visit on a single day	(i) Single diagnosis	A patient went to see a primary care doctor and received a single diagnosis.	01/01/2019^b^: Influenza
	(ii) Multiple diagnoses	A patient went to see a primary care doctor and received multiple diagnoses.	01/01/2018: Influenza | Pneumonia
Multiple visits on a single day	(iii) Single and same diagnosis for multiple visits	A patient went to see a primary care doctor and then got transferred to Emergency Room immediately.	01/01/2019: Acute myocarditis
			01/01/2019: Acute myocarditis
	(iv) Multiple diagnoses for multiple visits	A patient went to see a primary care doctor for flu. He also visited an endocrinologist for a routine follow-up for type II diabetes.	01/01/2019: Influenza with pneumonia | Acute myocarditis
			01/01/2019: Type II diabetes | Benign essential hypertension

^a^For better readability, the diagnosis codes are not listed

^b^01/01/2019 is a hypothetical date used for illustrative purpose

Therefore we considered the following three criteria when selecting seed patients for synthesizing patient medical records:

The patient medical records should contain the (i), (ii), and (iv) scenarios in Table 1. (iii) is nice to have, but not required for inclusion, because it is theoretically possible but practically extremely rare. In medicine, if a patient have multiple visits on the same day, it usually represents complicated situation with multiple diagnoses (e.g., primary and secondary diagnoses).

The selected patient must have both acute and chronic diseases on his or her medical records. We chose influenza and type II diabetes as representatives of acute and chronic diseases in this evaluation. Overall, we found that 3191 patients in the REP database meeting the first two criteria.

The patient medical records in the REP database have a wide range of length, in terms of total daily events. Figure 2 shows the distribution plot for the 3191 patients that satisfy both criteria (1) and (2). The mean and standard deviation of total daily events of these patients are 233.6 and 217.7, respectively. The patient count reaches its maximum when the total number of daily events is around 84. Accordingly, we selected four seed patients (the orange dots in Fig. 2), as their numbers of total daily events (9, 84, 224, and 458, respectively) spread out along the distribution.

#### Synthesis methods of patient medical records

We adopted three types of operations, namely deleting, updating and switching on the medical records of four selected seed patients at the level of daily event and event block (multiple daily events). Table 2 describes them in more details. In the context of sequence alignment, the operation of inserting in one sequence is equivalent to deleting in another sequence, so we only kept the latter. In the end, we synthesized 20 new patient medical records by applying one or more deleting, updating and switching operations, for each of the 4 seed patients. The second column of Tables 3 and 4 specified the operations we performed. The size of event block is determined by the maximum of (2, N/10), where N is the number of daily events for a seed patient.

**Table 2:** Operations of Deleting, Updating, and Switching on Daily Event and Multi-day Event Block

Operation	Level	
	Daily event	Event block
Deleting	Deleting a daily event	Deleting multiple consecutive daily events
Updating	Randomly changing a diagnosis in a daily event or randomly removing a diagnosis if the total number of diagnosis in a daily event is > 1	Changing a block of daily events
Switching	Switching all the diagnoses in two randomly selected daily events	Switching all the daily events between two selected daily event blocks of same length

**Table 3:** Similarity scores of pairwise global sequence alignments

ID	Operation	Seed Patient 1 (*N* = 9)			Seed Patient 2 (*N* = 84)			Seed Patient 3 (*N* = 224)			Seed Patient 4 (*N* = 458)		
		DTW	NWA	REF	DTW	NWA	REF	DTW	NWA	REF	DTW	NWA	REF
1	x	0.819	0.778	0.778	0.980	0.976	0.976	0.991	0.991	0.991	0.998	0.996	0.996
2	x x	0.597	0.556	0.556	0.976	0.952	0.952	0.987	0.982	0.982	0.994	0.991	0.991
3	u	0.852	0.852	0.852	*0.988*	*0.988*	*0.988*	0.996	0.996	0.996	0.996	0.996	0.996
4	u u	0.714	0.714	0.714	0.952	0.952	0.952	0.984	0.984	0.984	0.993	0.993	0.993
5	s	0.556	0.556	0.556	0.952	0.952	0.952	0.991	0.991	0.991	0.991	0.991	0.991
6	s s	0.286	0.286	0.175	0.905	0.905	0.905	0.988	0.988	0.988	0.983	0.983	0.983
7	x u	0.556	0.556	0.556	0.964	0.952	0.952	0.988	0.987	0.987	0.993	0.991	0.991
8	x s	0.611	0.556	0.556	0.929	0.929	0.929	0.978	0.973	0.973	0.989	0.987	0.987
9	u s	0.457	0.457	0.457	0.929	0.929	0.929	0.981	0.981	0.981	0.987	0.987	0.987
10	x u s	0.363	0.289	0.289	0.905	0.905	0.905	0.969	0.964	0.964	0.984	0.983	0.983
11	X	0.590	0.556	0.556	0.869	0.810	0.810	0.877	0.804	0.804	0.821	0.808	0.808
12	X X	0.179	0.111	0.111	0.702	0.667	0.667	*0.708*	*0.625*	*0.625*	0.657	0.633	0.633
13	U	0.667	0.667	0.667	0.821	0.821	0.821	0.832	0.832	0.832	0.831	0.831	0.831
14	U U	0.551	0.551	0.551	0.786	0.786	0.786	0.711	0.709	0.709	0.695	0.695	0.695
15	S	0.401	0.333	0.160	0.637	0.631	0.631	0.729	0.700	0.679	0.623	0.622	0.622
16	S S	*0.078*	*0.000*	*−0.269*	0.319	0.310	0.310	0.405	0.393	0.351	0.278	0.266	0.262
17	X U	0.185	0.185	0.185	0.702	0.676	0.676	0.679	0.668	0.668	0.716	0.704	0.704
18	X S	−0.204	−0.289	−0.333	0.539	0.530	0.530	0.577	0.552	0.495	0.509	0.501	0.474
19	U S	−0.204	−0.204	−0.204	0.526	0.518	0.518	0.689	0.685	0.664	0.646	0.640	0.636
20	X U S	−0.530	− 0.530	− 0.530	0.611	0.592	0.592	0.571	0.536	0.528	0.627	0.624	0.624

a. ID is the synthetic patient index. N is the number of daily events in a seed patient sequence

b. DTW, NWA and REF refer to as Dynamic Time Warping, Needleman-Wunsch Algorithm, and baseline reference, respectively

c. The lower case letters “x”, “u”, and “s” denote deleting, updating and switching a daily event, respectively. The upper case letters “X”, “U”, and “S” stand for deleting, updating and switching multi-day events (event block)

**Table 4:** Similarity scores of pairwise local sequence alignments

ID	Operation	Seed Patient 1 (*N* = 9)					Seed Patient 2 (*N* = 84)					Seed Patient 3 (*N* = 224)					Seed Patient 4 (*N* = 458)				
		DTWL		SWA		REF	DTWL		SWA		REF	DTWL		SWA		REF	DTWL		SWA		REF
		C	S_n_	C	S_n_	C (S_n_)	C	S_n_	C	S_n_	C (S_n_)	C	S_n_	C	S_n_	C (S_n_)	C	S_n_	C	S_n_	C (S_n_)
1	x	*1.000*	*0.819*	*1.000*	*0.778*	*0.667*	1.000	0.980	1.000	0.976	0.738	1.000	0.991	1.000	0.991	0.571	1.000	0.998	1.000	0.996	0.500
2	x x	1.000	0.597	1.000	0.556	0.333	1.000	0.976	1.000	0.952	0.452	1.000	0.987	1.000	0.982	0.509	1.000	0.994	1.000	0.991	0.511
3	u	1.000	0.852	1.000	0.852	0.444	1.000	0.988	1.000	0.988	0.964	1.000	0.996	1.000	0.996	0.741	1.000	0.996	1.000	0.996	0.917
4	u u	1.000	0.714	1.000	0.714	0.333	*1.000*	*0.952*	*1.000*	*0.952*	*0.667*	1.000	0.984	1.000	0.984	0.549	1.000	0.993	1.000	0.993	0.502
5	s	0.778	0.556	0.778	0.556	0.556	0.976	0.976	0.976	0.976	0.976	1.000	0.991	1.000	0.991	0.665	1.000	0.991	1.000	0.991	0.472
6	s s	1.000	0.286	1.000	0.286	0.222	1.000	0.905	1.000	0.905	0.333	1.000	0.988	1.000	0.988	0.969	0.998	0.985	0.998	0.985	0.469
7	x u	0.889	0.667	0.889	0.667	0.556	1.000	0.964	1.000	0.952	0.405	1.000	0.988	1.000	0.987	0.411	1.000	0.993	1.000	0.991	0.541
8	x s	1.000	0.611	1.000	0.556	0.444	1.000	0.929	1.000	0.929	0.369	1.000	0.978	1.000	0.973	0.652	1.000	0.989	1.000	0.987	0.526
9	u s	1.000	0.457	1.000	0.457	0.333	1.000	0.929	1.000	0.929	0.893	1.000	0.981	1.000	0.981	0.487	1.000	0.987	1.000	0.987	0.373
10	x u s	0.444	0.400	0.444	0.400	0.222	1.000	0.905	1.000	0.905	0.560	1.000	0.969	1.000	0.964	0.335	1.000	0.984	1.000	0.983	0.648
11	X	0.778	0.778	0.778	0.778	0.778	1.000	0.869	1.000	0.810	0.560	*1.000*	*0.877*	*0.804*	*0.804*	*0.804*	0.891	0.891	0.891	0.891	0.891
12	X X	0.333	0.333	0.333	0.333	0.333	1.000	0.702	1.000	0.667	0.619	1.000	0.708	1.000	0.625	0.509	1.000	0.657	1.000	0.633	0.618
13	U	*0.667*	*0.667*	*0.667*	*0.667*	*0.667*	1.000	0.821	1.000	0.821	0.762	0.835	0.835	0.835	0.835	0.835	0.893	0.893	0.893	0.893	0.893
14	U U	1.000	0.551	1.000	0.551	0.222	0.952	0.826	0.952	0.826	0.821	1.000	0.711	1.000	0.709	0.634	1.000	0.695	1.000	0.695	0.445
15	S	*0.778*	*0.401*	*0.333*	*0.333*	*0.333*	0.893	0.714	0.893	0.708	0.548	1.000	0.729	0.862	0.711	0.545	0.838	0.650	0.838	0.650	0.478
16	S S	0.556	0.389	0.556	0.333	0.222	0.690	0.500	0.690	0.500	0.452	0.728	0.463	0.728	0.460	0.335	*0.782*	*0.417*	*0.782*	*0.408*	*0.389*
17	X U	0.556	0.556	0.556	0.556	0.556	1.000	0.702	0.679	0.679	0.679	1.000	0.679	1.000	0.668	0.317	0.939	0.756	0.939	0.745	0.535
18	X S	0.222	0.222	0.222	0.222	0.222	1.000	0.539	1.000	0.530	0.262	1.000	0.577	0.821	0.561	0.362	0.734	0.569	0.734	0.567	0.476
19	U S	0.222	0.222	0.222	0.222	0.111	1.000	0.526	0.833	0.518	0.310	1.000	0.689	1.000	0.685	0.625	1.000	0.646	1.000	0.640	0.498
20	X U S	0.222	0.222	0.222	0.222	0.111	0.857	0.706	0.857	0.706	0.571	0.795	0.608	0.795	0.598	0.576	0.893	0.731	0.893	0.731	0.618

a. ID is the synthetic patient index. N is the number of daily events in a seed patient sequence

b. DTWL, SWA and REF refer to as modified Dynamic Time Warping for Local alignment, Smith-Waterman Algorithm, and baseline reference, respectively. C is the coverage of the seed patient sequence aligned to a synthetic patient sequence. S_n_ is normalized highest alignment score (i.e., the highest alignment score divided by N). C (S_n_) denotes that S_n_ = C

c. The lower case letters “x”, “u”, and “s” denote deleting, updating and switching a daily event, respectively. The upper case letters “X”, “U”, and “S” stand for deleting, updating and switching multi-day events (event block)

### Implementation of sequence alignment algorithms

We implemented DTW, NWA and SWA in python and the function module for each algorithm consists of two components: (1) Calculation of accumulated score matrix ***A***_*(n + 1) x (m + 1)*_ (2) Tracking back to identify an optimal alignment path. In addition, we also implemented a modified algorithm of dynamic time warping for local sequence alignment (DTWL) based on SWA.

More specifically, given two temporal sequences of medical events X ([X_1_, X_2_, …, X_i_, …, X_n_]) and Y ([Y_1_, Y_2_, …, Y_j_, …, Y_m_]), DTWL calculates an accumulated score matrix ***A***_*(n + 1) x (m + 1)*_ by updating the matrix element A_i, j_ according to the following equation,

4 $$ {A}_{i,j}=\Big\{{\displaystyle \begin{array}{cc}0& i=0\mathrm{or}j=0\\ {}\max \left(s\left({X}_i,{Y}_j\right)+{A}_{i-1,j-1},s\left({X}_i,{Y}_j\right)+{A}_{i-1,j},s\left({X}_i,{Y}_j\right)+{A}_{i,j-1},0\right)& i>0,j>0\end{array}}\operatorname{} $$

Where s (X_i_, Y_j_) denotes the similarity between two elements X_i_ and Y_j_ in the sequence of X and Y, and is calculated using a predefined scoring system as shown in Fig. 1(B).

During the calculation of accumulated score matrix, DTWL sets the matrix element with negative accumulated score to zero and make them invisible. After that, DTWL tracks back from the matrix element with the highest score until encountering zero to identify the optimal alignment path.

### Metrics for patient similarity

We adapted the scoring system commonly used in the biological sequence alignment shown in Fig. 1(B) to measure the similarity between two aligned daily events. In this scoring system, the score of matching is set to 1 as a reward. It also assigns the same score of − 1 to both mismatching and gap situations as a penalty. For two daily events (X and Y) involving multiple codes, we used Jaccard index J(X,Y) to measure their similarity s(X,Y) as follows,

5 $$ s\left(X,Y\right)=2J\left(X,Y\right)-1 $$

6 $$ J\left(X,Y\right)=\frac{\left|X\cap Y\right|}{\left|X\cup Y\right|} $$

We also penalized similarity between an original daily event in a patient sequence and an extra daily event inserted into another patient sequence by DTW or DTWL by setting score range between −1 (mismatching) and 0 (matching). In other words, the similarity s(X,Y) between them is defined as,

7 $$ s\left(X,Y\right)=J\left(X,Y\right)-1 $$

For global sequence alignments, we calculated the similarity score of aligned sequences by summing all the similarity scores s(X,Y) of aligned daily events. Due to the variation of daily event number in patient sequences, we further normalized the similarity score of aligned sequences by dividing the total number of daily events in the seed patient sequence. We used S_n_ to denote the normalized similarity score of aligned sequences.

For local sequence alignments, we calculated the normalized similarity score (S_n_) and coverage (C) of the longest aligned subsequences between seed patient and synthetic patient. S_n_ is the summation of the similarity scores s(X,Y) of daily events in the aligned subsequences and then is divided by the total number of daily events in the seed patient sequence. C is coverage of the seed patient sequence aligned to the synthetic patient sequence. Specifically, C is the ratio of the number of daily events in the seed patient sequence aligned to a synthetic patient sequence and the total number of daily events in the seed patient sequence.

## Results

### Pairwise global sequence alignment results

We synthesized 80 (4 × 20) patient medical records by performing the operations of deleting, updating and switching a daily event or a multi-day event block on the four seed patient records. We then performed global sequence alignment between each seed patient and each synthetic patient.

Table 3 lists the similarity scores of pairwise global sequence alignments from DTW and NWA on top of the medical records of each of the four seed patients and those of their corresponding synthetic patients. The results from DTW and NWA are compared with baseline references (REF).

We found that the similarity scores of DTW alignments were as good as, or even better than those of reference alignments. Particularly 47 out of 80 alignments made by DTW had even higher similarity scores than reference alignments. In addition, DTW alignments were better than NWA alignments on 46 cases out of 80, with the rest 34 cases having the equal similarity scores from both algorithms.

The NWA alignments also received better similarity scores than reference alignments − 11 out of 80 NWA alignments had superior similarity scores than reference alignment while the rest 69 had the same distance scores as reference alignment.

### Pairwise local sequence alignment results

After synthesizing 20 patient medical records for each out of 4 seed patients, we also performed local sequence alignment between medical records of each seed patient and each synthetic patient with DTWL and SWA to identify the longest aligned subsequences. We then calculated their similarity scores (S_n_) and coverage (C) for each pair of the longest aligned patient sequences. The results are shown in Table 4, together with baseline references (REF). Since C and S_n_ from baseline references are identical, we only show one of them in Table 4. It can be found that both coverage and similarity scores of DTWL alignments were as good as, or even better than those of reference alignments. Particularly 71 out of 80 alignments made by DTWL had even larger coverage than reference alignments and 70 out of 80 DTWL alignments had higher similarity scores than reference alignments. In addition, DTWL alignments were better than SWA alignments. More specially, 6 DTWL alignments showed larger coverage and higher similarity scores than SWA alignments. 30 out of 80 DTWL alignments had the equal coverage but better similarity scores than SWA. DTWL and SWA gave the equal coverage and similarity scores for the rest 44 cases.

NWA alignments also received better coverage and similarity scores than reference alignments. 69 (or 68) out of 80 NWA alignments had superior coverage (or similarity scores) than reference alignment while the rest 11 (or 12) had the same coverage (or similarity scores) as reference alignment.

## Discussion

**Fig. 3:**
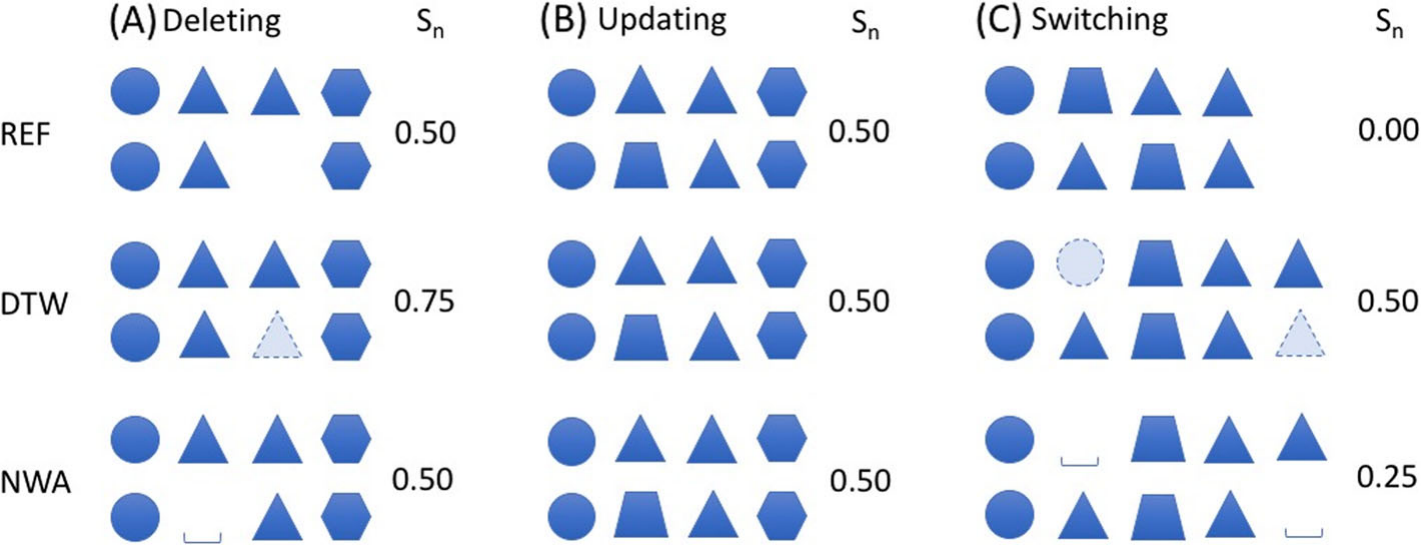
Scenarios of global sequence alignment: (**a**) Deleting, (**b**) Updating, and (**c**) Switching. REF, DTW, and NWA refer to as reference alignment, alignment with Dynamic Time Warping, and alignment with Needleman-Wunsch Algorithm, respectively. In each pair, seed sequence is listed on the top and aligned synthetic sequence is listed on the bottom. The similarity scores (S_n_) between seed sequence and synthetic sequence are also listed on the right side of each pair

**Fig. 4:**
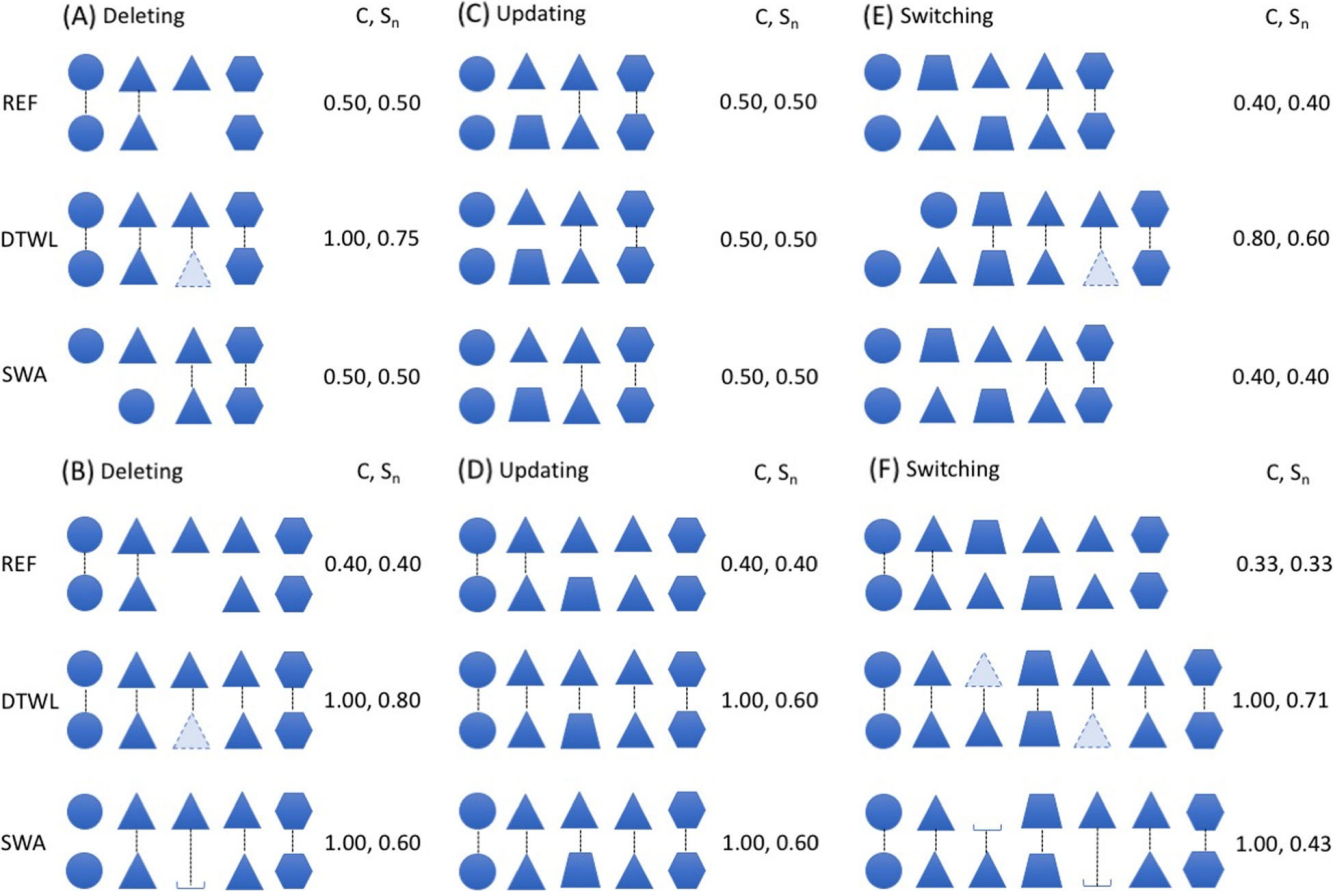
Scenarios of local sequence alignment: (**a**, **b**) Deleting, (**c**, **d**) Updating, and (**e**, **f**) Switching. REF, DTWL and SWA refer to as reference alignment, alignment with modified Dynamic Time Warping for Local alignment, and alignment with Smith-Waterman Algorithm, respectively. In each pair, seed sequence is listed on the top and aligned synthetic sequence is listed on the bottom. The coverage (C) and similarity scores (S_n_) between seed sequence and synthetic sequence are also listed on the right side of each pair

We carefully examined the raw global and local alignment results from 4 × 20 sequence pairs and noticed some subtle differences. We drew some cartoons in Figs. 3 and 4 to illustrate and discuss global alignments and local alignments, respectively.

### Pairwise global sequence alignment results

In Fig. 3(a), the reference alignment had a deletion, compared to the seed sequence, thus it received a similarity score of 0.50. NWA was able to insert a gap spot in the synthetic sequence for better alignment. The resulting alignment still received a similarity score of 0.50. However, the gap spot was inserted at a different position compared to reference alignment, which suggests there might be more than one alignment solutions. In contrast, DTW was able to stretch the synthetic sequence and insert a triangle daily event in the right position, so that the alignment result was identical to the seed sequence, which led to a similarity score of 0.75. As shown in Table 3 (the 1st, 2nd, 11th, and 12th rows), among 16 alignments between 4 seed patients and 4 synthetic patients created by only deleting operations, similarity scores of NWA alignments were the same as those of reference alignments. However, 15 out of 16 DTW alignments obtained higher similarity scores than NWA alignments, for example, the alignment between the 3rd seed patient and the 12th synthetic patient. This high similarity scores can be attributed to the fact that DTW uses the adjacent event to fill a gap position in a sequence.

The reference alignment shown in Fig. 3(b) had a daily event updating that the 2nd triangle daily event was replaced by a trapezoidal daily event. Thus, the distance score of the reference alignment was 0.50. Both DTW and NWA created the same alignments as the reference alignment. Among 16 alignments between seed patients and synthetic patients from only updating operations (the 3rd, 4th, 13th, and 14th rows in Table 3), 15 DTW or NWA alignments were identical to the reference alignments, for instance, the alignment between the 2nd seed patient and the 3rd synthetic patient.

In Fig. 3(c), the reference alignment contained a switch of two adjacent events (the triangle and the trapezoidal) and the corresponding similarity score was 0.0. NWA inserted a gap into both the seed sequence and synthetic sequence. The new sequence therefore became more similar to the seed sequence than the reference alignment (3 identical aligned daily events out of 5 vs. 2 identical aligned daily events out of 4). NWA alignment received a similarity score of 0.25. Similarly, DTW added a circle event into the seed sequence and a triangle event in the synthetic sequence, which generated a new sequence with 4 identical aligned daily events. DTW alignment had the highest similarity score (0.50). This also explains that 8 out 16 DTW alignments between seed patients and synthetic patients from switching operation (In Table 3) had higher similarity scores than NWA and reference alignments. 6 out of 16 NWA alignments were also better than reference alignments.

### Pairwise local sequence alignment results

In Fig. 3(a), the reference alignment contains the first two daily events due to a deletion of the 3rd daily event in the seed sequence. The coverage and similarity score of the reference alignment are 0.50. SWA aligned the last two daily events and had the same coverage and similarity score as the reference alignment, implying that multiple alignment solutions might exist. However, DTWL inserted a triangle daily event in the right position, thus the new sequence was identical to the seed sequence. DTWL alignment had the highest coverage (1.00) and similarity score (0.75). The typical example shown in Table 4 is the alignment between the 3rd seed patient and the 11th synthetic patient. The seed sequence in Fig. 3(b) had one more triangle daily event than that in Fig. 3(a). Thus, the reference alignment could be alignment of either the first two daily events or the last two daily events. Its coverage and similarity score are 0.40. Both SWA and DTWL made a full coverage alignment by inserting a gap or triangle daily event in the middle position. Due to the inserted triangle daily event, the similarity score of DTWL alignment is 0.80, which is higher than that of SWA alignment (0.60). In Table 4, among 16 alignments between 4 seed patients and 4 synthetic patients created by only deleting operations, 13 DTWL alignments and 12 SWA alignments performed better than corresponding reference alignments in terms of coverage and similarity scores. 11 DTWL alignments received higher similarity scores than SWA alignments while they both had a full coverage of 1.00.

The synthetic sequences in Fig. 3(c) and (d) had a trapezoidal daily event to replace a triangle daily event in the seed patient. In Fig. 3(c), both DTWL and SWA created the same alignments as the reference alignment. They all had identical coverage and similarity scores (0.50). Three similar cases can be found in Table 4, for example, the alignment between the 1st seed patient and the 13th synthetic patient. In Fig. 3(d), there are two equal options for the reference alignment: the alignment of the first two daily events or the alignment of the last two daily events. Only the reference alignment with the first two daily events is shown in Fig. 3(d). The coverage and similarity scores of reference alignments are 0.40. DTWL and SWA alignments had a full coverage (1.00) and identical similarity scores (0.60). We found that among 16 alignments between seed patients and synthetic patients from only updating operations (the 3rd, 4th, 13th, and 14th rows in Table 4), 12 DTWL and SWA alignments received a full coverage and equal same similarity scores, for example, the alignment between the 2nd seed patient and the 4th synthetic patient.

The reference alignments in Fig. 3(e) and (f) had a switch of two adjacent events (the triangle and the trapezoidal). In Fig. 3(e), the reference alignment contained the last two daily event and its coverage and similarity score are 0.40. SWA aligned a triangle daily event and a hexagonal daily event, so that SWA alignment received coverage and a similarity score of 0.50. DTWL stretched the synthetic sequence and inserted a triangle daily event in the right position. DTWL alignment had 4 daily events and received highest coverage (0.80) and similarity score (0.60). The similar situation in Table 4 is the alignment between the 1st seed patient and the 15th synthetic patient. In Fig. 3(f), the first or last two daily events can be aligned as the reference alignment. The coverage and similarity scores of the reference alignment are 0.33. Both DTWL and SWA had coverage of 1.00 due to the insertion of a daily event and gap spot while the similarity (0.71) of DTWL alignment is higher than that (0.43) of SWA alignment. In Table 4, among 16 alignments between seed patients and synthetic patients from switching operation, 14 DTWL alignments and 13 SWA alignments received better coverage and similarity scores than reference alignments. The coverage of 14 DTWL alignments were identical to the corresponding SWA alignments. Six DTWL alignments had higher similarity scores than SWA alignments.

### Limitations

This study for sure has several limitations, not limited to the following:

We only used diagnosis codes in our experiments. Other medical events such as demographics, procedures, medications, and clinical notes were not considered. We would like to incorporate more other medical event types for more comprehensive evaluation of sequence alignment algorithms in future, once we can infer the dependency between diagnosis and the other event types when synthesizing simulated patient medical records that still reflect reality, or when we can afford more expensive evaluation by physicians.

Secondly, we only used a limited number of operations to create synthetic patient records that reflect real-world situations in this study. We carefully selected 4 seed patients and created 20 synthesized patient medical records for each of them. This was driven by our goal of performing an objective and detailed 360-degree examination. This small size does not cover all the complex situations in large EHR database. After this, we could perform a much larger scale evaluation with confidence and precision.

Last but not the least, we used self-defined scoring system to quantitatively evaluate sequence alignment results. This scoring system penalizes mismatching and gap equally and also penalizes elements inserted by DTW and DTWL. We plan to design and test different scoring systems for evaluating sequence alignments. For example, a scoring system treats acute and chronic diseases differently by incorporating some knowledge base.

## Conclusions

A full consideration of temporal sequence information and aligning medical event sequences properly is fundamental for precise patient similarity calculation, since medicine is about providing patients the right diagnosis and treatments at the right timing. In this study, we synthesized patient medical records using a set of synthesis operations on top of real patient medical records from a large real-world EHR database. Then we tested two cutting-edge sequence alignment methods, dynamic time warping (DTW) and Needleman-Wunsch algorithm (NWA), and their corresponding versions for local alignments, modified DTW for Local alignment (DTWL) and Smith-Waterman algorithm (SWA), for the purpose of patient medical records alignment, in order to understand their strengths and limitations. We found that sequence alignment is very necessary for fully reserving the temporal sequence information in patient medical records. In addition, DTW (or DTWL) seemed to align better and identify more similarities between patient medical records than NWA (or SWA). But DTW, NWA, DTWL, and SWA performed better than the reference alignment. Our evaluation work could provide timely and valuable information on the strengths and weakness of these sequence alignment methods for the fast-growing areas of patient similarity calculation.

## Data Availability

The real-world EHR database can be accessed via the REP website (https://rochesterproject.org) upon reasonable request due to data privacy or other restrictions.

## Abbreviations

DTW: Dynamic time warping
DTWL: Modified DTW for Local alignment
EHR: Electronic health record
ICD-9-CM: International classification of diseases, ninth revision, clinical modification
NWA: Needleman-Wunsch Algorithm
REF: Baseline reference
REP: Rochester epidemiology project
SWA: Smith-Waterman Algorithm
